# The relationship between internalized weight stigma and physical and mental health-related quality of life in a large sample of women: a structural equation modeling analysis

**DOI:** 10.1007/s40519-023-01582-z

**Published:** 2023-06-21

**Authors:** Andrea Zagaria, Silvia Cerolini, Edoardo Mocini, Caterina Lombardo

**Affiliations:** 1 Department of Psychology, Sapienza University of Rome, Rome, Italy; 2 Department of Experimental Medicine, Sapienza University of Rome, Rome, Italy

**Keywords:** Weight stigma, Internalized stigma, Weight bias, Weight stereotypes, Mental health, Quality of life

## Abstract

**Purpose:**

Health-related quality of life (HRQOL) refers to an individual's perception of their physical and mental health status over time**.** Although emerging evidence has documented a negative association between weight stigma (i.e., negative weight-related attitudes and beliefs towards individuals with overweight or obesity) and mental HRQOL, its influence on physical HRQOL still needs to be fully clarified. This study aims to investigate the impact of internalized weight stigma on mental and physical HRQOL by employing a structural equation modeling (SEM) approach.

**Methods:**

The Short Form Health Survey 36 (SF-36) and the Weight Bias Internalization Scale (WBIS) were administered to a sample of 4450 women aged 18–71 (M_age_ = 33.91 years, SD = 9.56) who self-identified in a condition of overweight or obesity (M_BMI_ = 28.54 kg/m^2^; SD = 5.86). Confirmatory factor analysis (CFA) was conducted to assess the dimensionality of the scales before testing the proposed structural model.

**Results:**

After establishing the adequacy of the measurement model, SEM results revealed that internalized weight stigma was significantly and negatively associated with both mental (β = − 0.617; p < 0.001) and physical (β = − 0.355, p < 0.001) HRQOL.

**Conclusion:**

These findings offer additional support to prior research by confirming the association between weight stigma and mental HRQOL. Moreover, this study contributes to the existing literature by strengthening and extending these associations to the physical HRQOL domain. Although this study is cross-sectional in nature, it benefits from a large sample of women and the use of SEM, which offers advantages over traditional multivariate techniques, e.g., by explicitly accounting for measurement error.

*Level of evidence*: Level V, descriptive cross-sectional study.

## Introduction

Weight stigma may be defined as negative weight-related attitudes and beliefs towards individuals with a condition of overweight or obesity [1]. Weight stigma encompasses both external and internal manifestations [2]. Externally, it involves public stigma, which includes prejudices, stereotypes, and hostile attitudes or behaviors towards individuals because of their weight [2]. As a direct consequence of weight stigma experiences, individuals with a condition of overweight and obesity may internalize public weight stigma, applying these weight-based negative attitudes and stereotypes to themselves [3]. External weight stigma may be expressed and endorsed by different sources such as peers, healthcare professionals, coaches, media, and caregivers and may be negatively experienced by individuals with overweight and obesity [4, 5]. This may lead to real forms of weight-bullying and weight-based victimization in educational settings [6] and to discrimination and inequality (e.g., in recruitment, salary or treatment of staff) in the work environment [7]. Especially women, in addition to historically suffering from gender inequality, suffer from more weight-based discrimination in the workplace than men [8] and are stigmatized at lower weights than men [9]. Weight stigma is pervasive in Western culture and societies, especially in the media messages that convey unrealistic and perfectionist ideals of beauty [10] and condemn obesity as a consequence of laziness and lack of willpower [4]. Weight stigmatization is even common among health professionals and has been used to “motivate” patients to lose weight [11]. However, it produces the opposite effect: empirical findings suggest that stigmatizing obesity has negative behavioral consequences that may increase, rather than decrease, the weight of overweight individuals [12]. This effect may be explained through the minority stress model, with the activation of a “vicious cycle”, wherein weight stigma begets weight gain, perpetuating the condition of overweight/obesity and facilitating the internalization of weight stigma and the subsequent occurrence of negative outcomes [13, 14]. Not surprisingly, both obesity and weight bias internalization have been associated with multiple negative health-related outcomes including depression, anxiety, low self-esteem, poor body image, disordered eating, emotional difficulties, and suicidal ideation [15–18].

Health-related quality of life (HRQOL) is a multidimensional construct that captures an individual's subjective assessment of their physical and mental health status over time and its correlates, including health risks, medical conditions, functional abilities, social support, and socioeconomic status [19]. Recently, two meta‐analyses quantitatively synthesized the primary studies assessing the relationship between weight stigma and mental HRQOL among youth [2] and adults [20]. Their meta-analytic results confirmed that weight stigma (both external and internal) is associated with poorer mental HRQOL with medium to large effects. Particularly, Emmer and colleague [20] explored different aspects of adults’ mental health such as self‐esteem, well‐being, quality of life, life satisfaction, anxiety symptoms, depressive symptoms, body image dissatisfaction, disordered eating, and psychological distress. The study demonstrated a significant association between perceived weight stigma and decreased mental health. Interestingly, none of the hypothesized moderators (gender, age, adaptive coping strategies) had an impact on this association, except for body weight. Specifically, there was a stronger association between weight stigma and decreased mental health as BMI increased [20]. The lack of significant moderation by gender may seem somewhat counterintuitive considering that multiple studies have suggested a stronger association between weight stigma and HRQOL indicators in females [8, 9, 21, 22]. This stronger relationship may be attributed to the pervasive influence of the thin ideal in feminine beauty standards [20], underscoring the importance of further examining the role of weight stigma in the female population.

Even less is known about the association between weight stigma and physical HRQOL, with inconsistent findings reported thus far, as highlighted by a systematic review conducted by Papadopoulos and Brennan [23], particularly in community samples. Therefore, additional studies are needed to elucidate the extent to which self-related weight stigma may impact this domain.

In light of all the above, the present cross-sectional study aims to confirm and extend previous findings regarding the association between internalized weight stigma and physical and mental HRQOL among a large sample of Italian women reporting a condition of overweight or obesity. Based on previous research on mental HRQOL [2, 20], we hypothesised that women who report high levels of internalized weight stigma will exhibit lower levels of both mental and physical HRQOL.

## Methods

### Procedure

Participants voluntarily completed an online and anonymous survey hosted by the Qualtrics platform (https://www.qualtrics.com/). The survey lasted about 10 min and was advertised through the main social network platforms and by word of mouth. Participants fulfilled the inclusion criteria to participate in the study if: (1) signed informed consent; (2) were women aged 18 years or over; and (3) perceived themselves as being in a condition of overweight or obesity, a prerequisite for ensuring consistency with the statements of the Weight Bias Internalization Scale [3]. Specifically, after providing informed consent, a dichotomous screening question asked participants whether they selfperceived being in a condition of overweight or obesity. Individuals who classified themselves as normal-weight were excluded from the analyses of the current study. The first page of the survey contained a detailed description of the study, and respondents could quit the survey at any point. All study procedures were carried out in accordance with the Declaration of Helsinki and its later amendments. The study protocol was reviewed and approved by the Institutional Review Board of the Department of Psychology, Sapienza University of Rome (prot. 0000798).

### Participants

Participants were 4450 women, aged 18–71 (M_age_ = 33.91 years, SD = 9.56), who self-identified in a condition of overweight or obesity. The mean BMI reported by the sample was 28.54 kg/m^2^ (SD = 5.86). The majority of participants (i.e., 39.3%) obtained a high-school diploma as the highest level of education, 29.5% a master’s degree, 22% a bachelor’s degree, 9.2% a PhD level degree/post-graduate specialization, and 1.9% a middle-school diploma. With respect to marital status, 68.1% were unmarried, 28.1% were married, 3.5% were divorced, and 0.3% were widowed.

### Instruments

#### Sociodemographic characteristics

An ad-hoc form was designed to collect data on sociodemographic variables including gender, age, the highest level of education, and marital status. Moreover, respondents reported their current height and weight, from which body mass index (BMI) based on self-reported data was computed, i.e., weight (kg) ÷ height^2^ (meters). Eventually, participants were asked whether they currently received pharmacological treatments for hypertension and type 2 diabetes.

#### Internalized weight stigma

The Weight Bias Internalization Scale (WBIS) [3] was employed to assess self-directed stigma and stereotypes about overweight and obesity. Items are rated on a 7-point Likert scale ranging from 1 (strongly disagree) to 7 (strongly agree) (e.g., “My weight is a major way that I judge my value as a person” and “I don't feel that I deserve to have a really fulfilling social life, as long as I'm overweight”). Items were summed, with higher scores indicating greater internalization of weight-related stigma. The Italian version of the WBIS was administered [24], which is composed of 9 items that formed a reliable unidimensional structure and demonstrated satisfactory convergent and criterion validity.

#### Mental and physical health-related quality of life

The Short Form Health Survey 36 (SF-36) [25] is a widely used HRQOL questionnaire consisting of eight scales yielding two summary measures: the mental component summary scores (MCS) and the physical component summary score (PCS). More specifically, the MCS is a multifaceted second-order dimension encompassing four key health concepts as first-order factors: (1) mental health (MH, i.e., psychological distress and wellbeing); (2) social functioning (SF, i.e., limitations in social activities); (3) vitality (VT, i.e., energy and fatigue); and (4) role-emotional (RE, i.e., limitations in regular daily activities due to emotional problems) [26]. Similarly, the PCS is a second-order dimension encompassing several first-order factors: (1) physical functioning (PF, i.e., limitations in physical activities due to health problems); (2) role physical (RP; i.e., limitations in daily activities due to physical health problems); (3) bodily pain (BP, i.e., intensity of bodily pain and discomfort); and (4) general health (GH, i.e., general health perceptions) [26]. Each subscale is transformed into a 0–100 scale, where lower scores reflect lower mental and physical HRQOL. The Italian version of the scale demonstrated solid psychometric properties confirming the hypothesized dimensions of health seen in United States data [27].

### Data analysis

Data were analysed through IBM SPSS version 23 (IBM Corporation, Armonk NY; USA) and Mplus version 8.6 [28].

The impact of internalized weight stigma on mental and physical HRQOL was examined within the structural equation modeling (SEM) framework. With the aim to maximize the reliability and the proportion of true-score variance to unique variance, as well as to reduce sources of sampling error, the number of parameters estimates and the likelihood of correlated residuals (see [29, 30] for an extensive discussion), latent factors were defined using a parceling strategy. In light of the unidimensional structure of the WBIS [24], following Matsunaga’s suggestions [31], three three-item parcels were created by summing and allocating the items based on the corrected item-total correlations (i.e., the so-called balancing approach) [29]. In regard to the MCS and PCS, both are conceptualized as second-order dimensions consisting of four correlated factors. Accordingly, each parcel was made up of observed indicators that loaded on the same first-order factor. This approach, known as homogenous parceling, is recommended when a second-order construct encompasses multiple first-order factors [30].

Preliminarily to model testing, descriptive statistics and missing data were examined for each parcel. Skewness and kurtosis were calculated, with values greater than |1| indicating non-negligible departures from the univariate normal distribution [32]. Moreover, we empirically tested whether missing data occurred completely at random through Little’s MCAR test [33]. These assumption checks guided subsequent decisions on parameter estimation and handling of missing data.

**Fig. 1:**
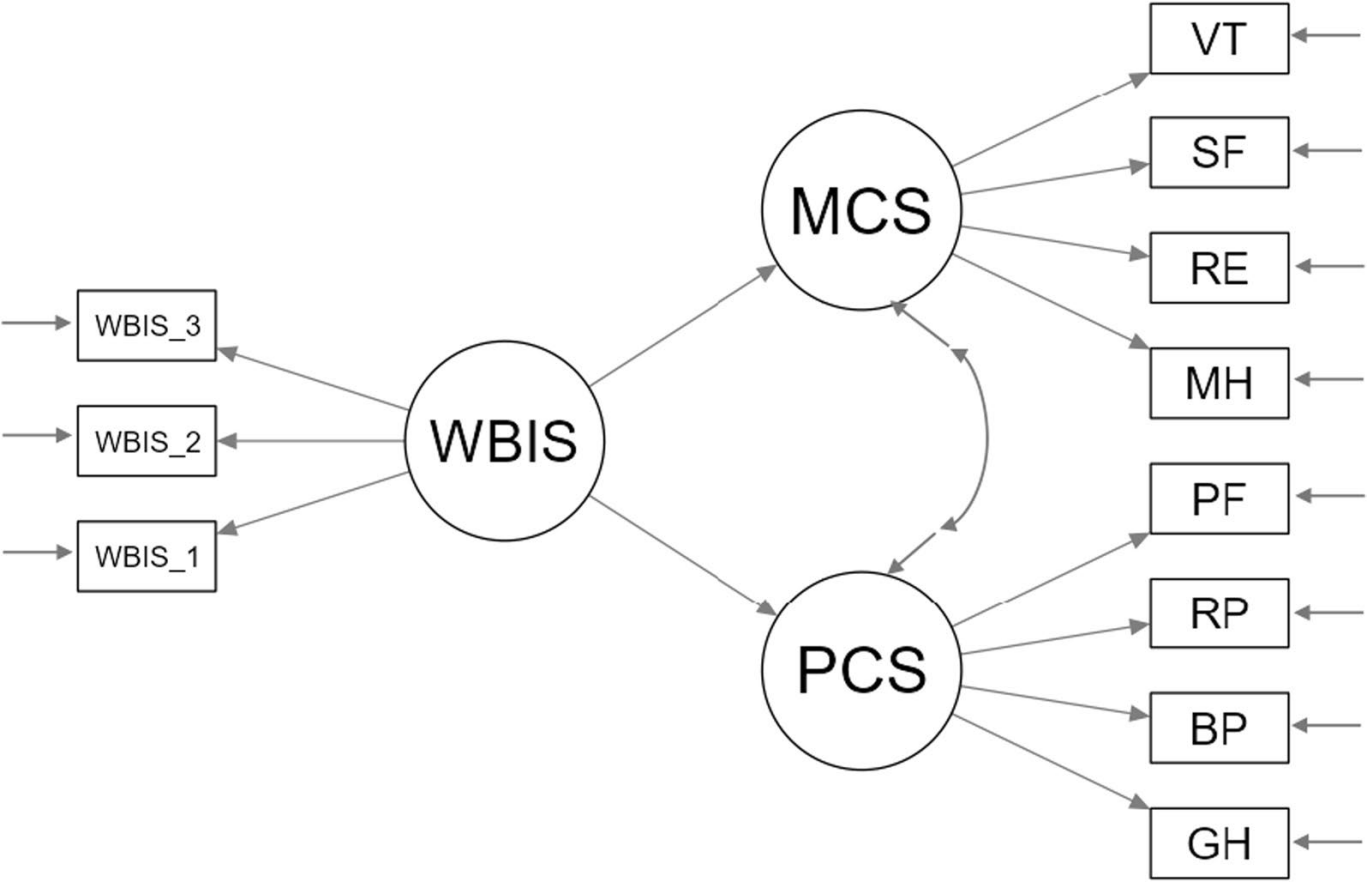
The posited SEM model. Latent variables were defined by parcels. Covariates (i.e., age, BMI, education, and therapies for hypertension/type 2 diabetes) were not depicted to avoid clutter. *WBIS* weight bias internalization scale, *MCS* mental component summary score, *PCS* physical component summary score, *MH* mental health, *SF* social functioning, *VT* vitality, *RE* role emotional, *PF* physical functioning, *RP* role physical, *BP* bodily pain, *GH* general health

A full SEM is characterised by two basic components: (1) the measurement model and (2) the structural model (e.g., [34]). As a first step, the measurement model was examined by conducting a confirmatory factor analysis (CFA) consisting of the hypothesized three latent constructs and their respective parcels as manifest indicators. The degree to which indicators of a construct converge and share a high proportion of variance in common was assessed using a comprehensive approach based on recommendations by Hair and colleagues [34]. A good convergence entails standardized loadings exceeding 0.5, average variance extracted (AVE) values greater than 0.5, and composite reliability exceeding 0.7 [34]. A model-based composite reliability coefficient (see [35]) was preferred to the standard Cronbach’s alpha since the latter may produce biased estimates of reliability when measures are not essentially tau-equivalent [36]. Furthermore, we specified an alternative model where all observed indicators loaded onto a single latent factor (i.e., Harman’s single-factor test) with the aim of evaluating the discriminant validity of the investigated constructs and potential issues of common method bias [37, 38]. As a second step, after establishing the validity of the measurement model, we tested our substantive SEM by specifying the structural coefficients depicted in Fig. 1, while accounting for several covariates (i.e., age, BMI, education, and pharmacological therapies).

In line with a multifaceted approach to the assessment of model fit, several indices were considered to evaluate the fit of the models to the observed data: the Root Mean Square Error of Approximation (RMSEA; < 0.08 indicates moderate fit) [39]; the Comparative Fit Index (CFI;  > 0.90 indicates acceptable fit) [40]; the Tucker Lewis index (TLI; > 0.90 indicates acceptable fit) [40]; and the standardized root mean square residual (SRMR;  < 0.08 indicates good fit) [40]. The χ2 likelihood ratio test was not taken into consideration due to its tendency to yield statistically significant values even with small model–data discrepancies in large samples [41].

## Results

### Preliminary analyses

Descriptive statistics for the observed variables (i.e., parcels) included in further models are reported in Table 1. With the exception of PF for which a considerable violation of normality was observed (we applied a square root transformation prior to further analyses, falling within the criterion range of ± 1) (see [42]), the study parcels presented minor departures from the univariate normal distribution. Accordingly, CFAs and structural equation models were analysed using robust maximum likelihood parameter estimates (MLR) with standard errors and chi-square test statistics robust to non-normality [28]. The MLR standard errors are calculated using a sandwich estimator, while the corrected chi-square test statistics are asymptotically equivalent to the Yuan-Bentler T2* test statistic [43]. By further exploring non-responses on each parcel, we observed that the missing data points occurred completely at random (Little’s MCAR: χ^2^(133) = 160.139, p > 0.05). Therefore, missing values were handled using the full information maximum likelihood approach (FIML) [44]; such an approach uses all available data points without listwise deletion and provides unbiased and efficient estimates under ignorable missing data conditions [45].

**Table 1:** Descriptive statistics and measurement model results

Construct	Descriptive statistics			Measurement model		
	Mean (SD)	Skewness	Kurtosis	Factor loading	Composite reliability	AVE
*Mental HRQOL*					0.853	0.597
VT	44.31 (17.78)	− 0.01	− 0.30	0.817		
SF	61.08 (24.93)	− 0.24	− 0.44	0.776		
RE	50.19 (42.82)	− 0.02	− 1.69	0.598		
MH	52.32 (18.68)	− 0.14	− 0.44	0.872		
*Physical HRQOL*					0.749	0.429
PF^a^	91.34 (12.11)	− 2.53^a^	8.92^a^	0.620		
RP	83.06 (27.45)	− 1.65	1.82	0.592		
BP	74.81 (22.78)	− 0.53	− 0.62	0.657		
GH	64.02 (19.82)	− 0.54	− 0.11	0.741		
*Internalized weight bias*					0.910	0.770
WBIS_Parcel_1	12.02 (4.78)	0.06	− 0.92	0.906		
WBIS_Parcel_2	14.35 (4.69)	− 0.60	− 0.46	0.871		
WBIS_Parcel_3	12.01 (4.51)	− 0.17	− 0.71	0.855		

All factor loadings are reported in a completely standardized metric and are statistically significant (p < .001). ^a^Prior to model testing, a square root transformation was applied for PF due to a substantial failure of normality [see 42]. *SD* standard deviation, *HRQOL* health-related quality of life, *AVE* average variance extracted, *MH* mental health, *SF* social functioning, *VT* vitality, *RE* role emotional, *PF* physical functioning, *RP* role physical, *BP* bodily pain, *GH* general health, *WBIS* weight bias internalization scale

### Measurement model

We first examined the dimensionality of the scales by means of confirmatory factor analysis (CFA). The CFA model positing three latent dimensions defined by their respective parcels as manifest indicators showed a good fit to the data: χ^2^ = 928.096 (41), p < 0.001, CFI = 0.959; TLI = 0.945; RMSEA = 0.070 (90% CI 0.066 to 0.074), SRMR = 0.040. The latent factors were significantly loaded by the intended indicators (ps < 0.001), with standardized loadings ranging from 0.592 to 0.906 (see Table 1). Each latent construct fulfilled the criterion for internal consistency, with composite reliability coefficients ranging from 0.749 to 0.910. Moreover, AVE values (i.e., the average percentage of variation explained among the items of a construct) ranged from 0.429 (PCS) to 0.770 (WBIS). As stated by Fornell and Larcker [35, p. 46], AVE is a “*more conservative measure than composite reliability*” and therefore “*on the basis of composite reliability alone, the researcher may conclude that the convergent validity of the construct is adequate*”. Accordingly, these estimates converged to indicate a satisfactory validity of the measurement model. Furthermore, Harman’s single-factor test demonstrated a very poor fit to the data, supporting the discriminant validity of the study variables and the absence of a substantial common method bias: χ^2^ = 11,191.955 (44), p < 0.001, CFI = 0.481; TLI = 0.351; RMSEA = 0.239 (90% CI 0.235 to 0.242), SRMR = 0.114.

### Structural model

After establishing the goodness of the measurement model, the structural paths graphically depicted in Fig. 1 were tested whilst controlling for the effects of several covariates including age, BMI, education, and therapies for hypertension and type 2 diabetes (i.e., 0 = no; 1 = yes). The effects of covariates that did not reach a marginal statistical significance (p > 0.10) were fixed to 0 in order to avoid overcontrol [46]. A chi-square difference test highlighted that constraining to 0 these paths did not worsen the model significantly: Δχ^2^(2) = 1.917 p > 0.05. The model exhibited an acceptable fit to the empirical data: χ^2^(83) = 1785.073, p < 0.001; CFI = 0.928; TLI = 0.905; RMSEA = 0.068 (90% CI 0.065 to 0.071), SRMR = 0.043. More specifically, internalized weight stigma was significantly and negatively associated with both mental (β = − 0.617; p < 0.001) and physical (β = − 0.355, p < 0.001) HRQOL. Overall, 39% of the variability of mental HRQOL and 28% of the variability of physical HRQOL were explained by the model.

## Discussion

This study aimed to confirm and extend previous findings supporting the association between internalized weight stigma and HRQOL. Particularly, physical and mental HRQOL were evaluated in a large sample of adult women reporting a condition of overweight or obesity. Results showed that the internalization of weight stigma was negatively associated with both mental and physical HRQOL indicators. That is, as hypothesised, those women reporting higher interiorization of stereotypes and negative attitudes regarding their overweight also referred to lower quality of life in the mental and physical HRQOL domains. The posited model explained a meaningful proportion of observed variance (39% of mental HRQOL and 28% of physical HRQOL) after controlling for the effects of potential confounding factors including age, BMI, education, and therapies for hypertension and type 2 diabetes. By applying Cohen's benchmarks [47] to assess the magnitude of these effects, we can classify them as substantial (> 26%), indicating a noteworthy capability of the model in predicting and explaining the HRQOL outcomes.

Results on mental HRQOL align with meta-analytic findings encompassing both youth [1] and adults [18]. Additionally, the results on physical HRQOL strengthen and expand the limited existing research conducted on community samples [19], contributing to our understanding of the paradoxical effects of internalized weight stigma. These findings are also consistent with studies involving pre-bariatric surgery patients with obesity, where high internalized weight stigma seems to predict impairments in HRQOL domains by diminishing self-esteem and increasing psychological distress [48]. Moreover, the current study corroborates evidence from the Italian context, which found a significant association between weight stigma and mental HRQOL indicators among clinical samples of individuals with overweight and obesity [24]. Furthermore, emerging findings from community samples in Italy underscore the prevalence of stigmatizing situations, experienced by 98% of individuals with obesity, particularly in public settings and from strangers [49].

Notably, the present study provides a comprehensive overview of the Italian scene by including a very large sample of over four thousand women who self-perceived in a condition of overweight or obesity. In addition to sample amplitude, which is a crucial factor for enhancing external validity and generalizability of the study findings, the present investigation employed a full SEM approach which has several advantages compared to traditional multivariate techniques such as ordinary least squares regression, e.g., by providing a flexible framework for examining linear relationships among multiple variables whilst simultaneously partialing out measurement error, a common issue in social sciences data that may lead structural coefficients to be either over- or underestimated (see [50, 51] for an extensive discussion).

The findings may suggest important clinical implications for clinicians and healthcare professionals who provide care for people affected by overweight and obesity (e.g., [52–54]). Extensive evidence consistently supports the presence of weight-biased attitudes and stereotypes among healthcare professionals [55, 56], which can inadvertently perpetuate the harmful cycle of weight stigma [13]. In light of the emerging association between weight stigma and HRQOL, healthcare professionals involved in the diagnosis and treatment of obesity may undergo specific training on weight stigma aimed at enhancing their awareness of the interconnectedness of these factors and equipping them with the knowledge and skills necessary to prevent the reinforcement of stigmatizing beliefs that could hinder patients' adherence to prescribed therapies (e.g., [23]). In clinical settings, the assessment of experienced, perceived, and internalized weight stigma may be integrated into evaluations of patients with obesity. This will facilitate the identification of areas for intervention that go beyond weight control and instead focus on how patients perceive the issue and its pervasive implications for their mental health (see [23] for a review). Furthermore, educational interventions or lifestyle interventions, especially in school settings or when targeting young individuals, should consistently address weight bias issues and stigma to prevent the perpetuation of misconceptions and challenge erroneous beliefs. This is particularly crucial for youth with higher BMI, as they are often targets of victimization from their peers, parents, and teachers [57].

Eventually, it is important to acknowledge several limitations of the present study. Firstly, our study was cross-sectional in nature, which precludes the possibility of establishing the directionality of the associations. Additionally, we relied on self-report questionnaires, which may be subject to social desirability bias, particularly regarding the construct of weight bias, where shame and embarrassment resulting from weight stigmatization play a crucial role, as suggested by the cyclic obesity/weight-based stigma model [13]. Future research should prioritize longitudinal and experimental studies, employing objective and validated measurements, to further investigate this domain. Furthermore, it is noteworthy that the current study exclusively focused on a large sample of women. Hence, future studies should be replicated in a more representative sample of the general population, enabling the exploration of potential gender differences in the relationship between weight bias and HRQOL. Lastly, additional unexplored factors should be taken into account to better understand the connections between weight bias and HRQOL, including psychopathological symptoms (such as depression and anxiety) and socio-demographic factors (such as income and occupation) (e.g., [23]). Future studies are warranted to consider these variables in order to elucidate the unique contribution of weight bias to HRQOL.

### What is already known on this subject?

Weight stigma, i.e., negative weight-related attitudes and beliefs towards individuals with overweight or obesity, is pervasive in Western culture and societies and has been associated with multiple negative health-related outcomes including depression, anxiety, low self-esteem, poor body image, disordered eating, emotional difficulties, and suicidal ideation. Although emerging evidence has documented a negative association between weight stigma and mental health-related quality of life (HRQOL), its impact on physical HRQOL is still yet to be fully clarified among community samples.

### What this study adds?

This investigation has notable strengths, including a large sample size (n = 4450) of women with a self-reported condition of overweight or obesity, which enhances the external validity and generalizability of the study findings. Moreover, the use of structural equation modeling (SEM) techniques provides advantages over traditional multivariate techniques by explicitly addressing measurement error, a common issue in social science data. Additionally, the study complements and expands upon previous limited findings on the physical HRQOL domain on community samples.

## Data Availability

The datasets generated during and/or analysed during the current study are available from the corresponding author upon reasonable request.
